# Rheological Characterization of Genipin-Based Crosslinking Pigment and O-Carboxymethyl Chitosan–Oxidized Hyaluronic Acid In Situ Formulable Hydrogels

**DOI:** 10.3390/polym16182615

**Published:** 2024-09-15

**Authors:** Ivo Marquis Beserra Junior, Débora de Sousa Lopes, Milena Costa da Silva Barbosa, João Emídio da Silva Neto, Henrique Nunes da Silva, Marcus Vinícius Lia Fook, Rômulo Feitosa Navarro, Suédina Maria de Lima Silva

**Affiliations:** 1 Postgraduate Program in Materials Science and Engineering, Department of Materials Engineering, Federal University of Campina Grande, Campina Grande 58429-900, PB, Brazilmilecost@hotmail.com (M.C.d.S.B.); henrique.nunes.silva.eng@gmail.com (H.N.d.S.);; 2 Department of Chemistry, State University of Paraíba, Campina Grande 58429-500, PB, Brazil; 3 Department of Materials Engineering, Federal University of Campina Grande, Campina Grande 58429-900, PB, Brazil

**Keywords:** post-surgical adhesion prevention, physical barriers, viscoelastic polymer hydrogels, rapid self-healing

## Abstract

The aim of this study was to develop a material capable of rapidly absorbing bodily fluids and forming a resilient, adhesive, viscoelastic hydrogel in situ to prevent post-surgical adhesions. This material was formulated using O-carboxymethyl chitosan (O-CMCS), oxidized hyaluronic acid (OHA), and a crosslinking pigment derived from genipin and glutamic acid (G/GluP). Both crosslinked (O-CMCS/OHA-G/GluP) and non-crosslinked hydrogels (O-CMCS/OHA) were evaluated using a HAAKE™ MARS™ rheometer for their potential as post-surgical barriers. A rheological analysis, including dynamic oscillatory measurements, revealed that the crosslinked hydrogels exhibited significantly higher elastic moduli (*G*′), indicating superior gel formation and mechanical stability compared to non-crosslinked hydrogels. The G/GluP crosslinker enhanced gel stability by increasing the separation between *G*′ and *G*″ and achieving a lower loss tangent (tan *δ* < 1.0), indicating robustness under dynamic physiological conditions. The rapid hydration and gelation properties of the hydrogels underscore their effectiveness as physical barriers. Furthermore, the O-CMCS/OHA-G/GluP hydrogel demonstrated rapid self-healing and efficient application via spraying or spreading, with tissue adherence and viscoelasticity to facilitate movement between tissues and organs, effectively preventing adhesions. Additionally, the hydrogel proved to be both cost effective and scalable, highlighting its potential for clinical applications aimed at preventing post-surgical adhesions.

## 1. Introduction

A viscoelastic material is a material that has the characteristics of both an elastic solid and a viscous fluid. From the point of view of the stress–strain relationship, in an elastic solid, the stress and strain are in phase, that is, they are in the same direction with a phase angle *δ* = 0, generating the possibility, within the elastic regime, of all deformation caused by stress to be completely recovered after the stress is removed. On the other hand, in a viscous fluid, stress and strain are 90° out of phase with each other, which makes any deformation resulting from applied stress irreversible. Thus, for a viscoelastic material, the phase angle would be between 0° and 90°, indicating that part of the deformation is recovered and part of the deformation remains after the external stress is removed [1,2].

In this aspect, a viscoelastic material will be characterized by an elastic or storage modulus, *G*′, and a viscous or loss modulus, *G*″, as components of its complex modulus, G*, according to Equation (1).

(1) $$G^{*}=G^{\prime }\pm iG^{\prime \prime }$$

With time within a given frequency range, *G*′ and *G*″ can be defined by relations such as those shown in Equations (2) and (3).

(2) $$G^{\prime }=G_{0}\cos\delta$$

(3) $$G\prime \prime =G_{0}\sin\delta$$

where $G_{0}=\frac{\tau_{ph}}{\gamma_{ph}}$, namely, the quotient of the amplitudes of stress and strain that are in phase, and *δ* defines the viscoelastic character of the material.

Since when 0≤tan⁡δ<1.0 the material behaves as a solid, it has a viscous character when tan⁡δ>1.0.

Hydrogels are three-dimensional polymeric networks with high water content that have received significant attention for biomedical applications, including tissue engineering and medical devices. Their ability to retain water, appropriate elasticity, and network structures make them ideal for imitating the natural extracellular matrix (ECM) of tissues [3,4,5].

One notable application of hydrogels is the prevention of post-surgical tissue adhesions [6,7,8,9,10,11,12]. Post-surgical adhesion is a common complication that frequently occurs after various types of surgeries [13], potentially affecting almost any part of the body and causing lifelong risks [14,15,16]. These adhesions not only negatively impact the quality of life of patients by causing chronic pain, secondary infertility in women, intestinal obstruction, dysphagia, and movement disorders but also can prolong surgery duration and hospital stays during revision surgeries, increasing the rate of major complications [11,17,18,19]. Thus, there is a growing need for the development of anti-adhesion barriers that can serve as effective physical barriers between injured tissues and adjacent organs in clinical practice.

Current commercial anti-adhesion materials are primarily applied in the form of solid films or powders, which form a sol-like barrier on the tissue surface [8,20,21,22,23]. However, these materials have significant limitations. In addition to being costly, most of them require preoperative preparation of the precursor solution, necessitating a sterile environment and additional surgical setup, thereby further increasing the overall cost of the procedure. Alternatively, pre-fabricated solutions of natural macromolecules tend to degrade spontaneously in aqueous environments, reducing their shelf life and increasing storage and transportation costs [24,25].

The development of biocompatible hydrogels for the prevention of post-surgical tissue adhesions represents a significant advancement in the biomedical field but still faces complex challenges. Studies have shown that hydrogels based on hyaluronic acid and chitosan are effective in creating physical and anti-inflammatory barriers critical for preventing adhesion formation following abdominal and gynecological surgeries [26,27,28]. Recently, research has advanced with the development of customized hydrogels, such as 3D-printed alginate scaffolds loaded with Avastin [29] and curcumin-based hydrogels to reduce intrauterine adhesions [30]. However, the need to balance the biocompatibility, durability, and functional efficacy of these materials, while considering individual physiological variables, makes the design of these hydrogels a multidisciplinary challenge. The increasing sophistication of these approaches underscores the critical role of these materials in enhancing surgical practices and minimizing post-operative complications. This highlights the ongoing need for innovation in creating effective and safe hydrogels. Consequently, developing hydrogels as barrier materials to prevent post-surgical tissue adhesions continues to be a major challenge in the biomedical field.

Sprayable or in situ formed hydrogels emerge as a promising alternative for the proposed application. Ready-to-use powder can be stored at room temperature for extended periods and utilized with designed delivery devices that distribute the powder uniformly and stably. An integrated clinical solution like this certainly optimizes surgical programs and reduces medical costs. In this study, we synthesized a rapidly gelling powder of O-CMCS/OHA, crosslinked with a pigment synthesized from the combination of genipin and glutamic acid (O-CMCS/OHA-G/GluP), for the formation of post-surgical barrier hydrogels. This powder was easily fabricated and rapidly hydrated. Additionally, all materials used in the preparation of the powder are components approved for clinical use. O-CMCS, hyaluronic acid, genipin, and glutamic acid are widely used in medical and pharmaceutical applications [31,32,33,34,35,36].

For this powder to be effective as a barrier material for preventing post-surgical adhesions, it must, in addition to being biodegradable and biocompatible, be capable of flowing viscously when subjected to shear (e.g., during spraying), allowing it to conform to and completely cover the target tissue. After application, the material should rapidly form a solid physical barrier that adheres to the tissue, does not delaminate, and remains solid in a minimally disturbed environment. The material must be able to flow between adjacent body structures while remaining adhered to the tissue, adjusting to the natural movement of the body.

Rheology can be successfully used to characterize all kinetics and mechanisms of gelation, as extensively discussed by Malkin and Derkach [37]. Dynamic oscillatory measurements using a range of frequencies at a constant temperature can be used to quantify gel strength. The technique can be used to determine the condition for gelation for a range of temperatures [2,38,39,40,41,42,43,44,45,46]. The increase in oscillatory frequency and level of concentration in gels caused a monotonic increase in both elastic and viscous moduli [47]. The gel point is defined by the frequency (or time) when *G*′ = *G*″ and, consequently, tan⁡δ=1.

It was shown that the viscoelastic properties of gelling material around the gelation time can be analyzed using an extended shear relation modulus expression with a functional form based on a product of power law and Debye Maxwell kernels [38,48,49]. Indeed, the sol–gel transition (SGT) is characterized by three viscoelastic domains: a pre-SGT domain (*t* < $t_{g}$), a critical gel CG domain (*t* = $t_{g}$), and a post-SGT domain (*t* > $t_{g}$). Analytical expressions associated with these three viscoelastic domains for the storage *G*′ and loss *G*″ moduli, which depend on both the viscoelastic time (or angular frequency) and the extent of the reaction, have been well established elsewhere [48,49].

In this study, hydrogels composed of O-CMCS/oxidized hyaluronic acid, crosslinked with a pigment synthesized from the combination of genipin and glutamic acid, were rheologically characterized to evaluate their effectiveness as post-surgical barrier materials.

## 2. Experimental Methods

### 2.1. Materials

Chitosan powder with an acetylation degree (AD) of 12% and a molecular weight (Mv) of 270 kDa, used in the synthesis of O-CMCS, was supplied by Northeastern Biomaterials Evaluation and Development Laboratory—Certbio (Campina Grande, PB, Brazil). Isopropanol, sodium hydroxide, monochloroacetic acid, and absolute ethanol, also employed in the synthesis of O-CMCS, were purchased from Sigma-Aldrich (Rio de Janeiro, Brazil). Hyaluronic acid was supplied by Farma Face Compounding Pharmacy (Campina Grande, Brazil). Ethylene glycol (EG) was purchased from Nuclear (São Paulo, SP, Brazil). Sodium periodate (NaIO_4_) was obtained from ACs Científica (São Paulo, SP, Brazil). Genipin and phosphate-buffered saline (PBS) (pH 7.2 at 25 °C) were acquired from Sigma-Aldrich (Rio de Janeiro, RJ, Brazil), and glutamic acid was purchased from Dinâmica (São Paulo, SP, Brazil).

### 2.2. Synthesis of O-Carboxymethyl Chitosan (O-CMCS)

For the synthesis of O-CMCS, the methodology adapted from Galdino et al. [50] was used. An alcoholic solution of chitosan was prepared with a concentration of 55.5 g/L and stirred magnetically for 30 min. Then, 4 mL of a sodium hydroxide solution (12.50 mol/L) was added to the chitosan solution, and the mixture was maintained under stirring for 24 h. Subsequently, a solution of monochloroacetic acid in isopropanol (3.97 mol/L) was slowly added to the mixture, and the stirring was maintained for another 24 h. After this period, 60 mL of absolute ethanol was added under magnetic stirring. The resulting solution was filtered and washed ten times with 80% ethanol, using 50 mL of the ethanol solution in each wash to completely remove the carboxymethyl chitosan salt. A final wash with absolute ethanol was performed to adjust the pH and remove residues until the pH value was close to 7. After filtration, the product was stored at room temperature (24 °C) for 12 h to dry in a desiccator. The obtained powder was pulverized in a mortar with the aid of liquid nitrogen and passed through a 25-mesh sieve, resulting in a O-CMCS powder with a mean particle size of less than 0.7 mm.

### 2.3. Synthesis of Oxidized Hyaluronic Acid (OHA)

Hyaluronic acid (HA) was dissolved in ultrapure water at room temperature (25 ± 1 °C) and oxidized with sodium periodate (NaIO_4_) based on adaptations of previously reported methodologies [51,52,53,54,55]. Briefly, 5 mL of 0.5 M NaIO_4_ was added to the previously prepared HA solution (10 mg/mL). The mixture was stirred mechanically at a constant speed of 2500 rpm for 2 h at 25 ± 1 °C and kept in the dark for 24 h. Subsequently, 1 mL of ethylene glycol was added to stop the oxidation reaction, and the solution was stirred for an additional hour. The solution was then transferred to a dialysis sacks (supplied by Sigma Aldrich, Rio de Janeiro, Brazil) and dialyzed for 3 days at 25 °C in a container with ultrapure water, with periodic water changes. The resulting OHA solution was frozen at −80 ± 2 °C and subjected to lyophilization at −45 °C for 72 h using a lyophilizer (Liotop model L108). The freeze-dried product was then pulverized in a mortar, passed through a 100-mesh sieve, and the resulting powder of OHA with a mean particle size of less than 0.1 mm was stored in a desiccator.

### 2.4. Synthesis of the Genipin–Glutamic Acid Crosslinker Pigment (G/GluP)

The G/GluP was synthesized using an adapted version of previously described methodologies [56,57,58]. Initially, a 7.0 mM glutamic acid (Glu) solution was prepared in water. In parallel, a genipin (G) solution was prepared in phosphate buffer at pH 7.0 with a concentration of 0.7 mM. Equal volumes of both solutions were then mixed in a sealed Erlenmeyer flask and maintained under magnetic stirring at 70 °C in a thermostatic bath for 5 h to form the crosslinker pigment (G/GluP). After the reaction, the solution was frozen at −80 ± 2 °C for 72 h and lyophilized for another 72 h. The obtained powder was then ground and passed through a 25-mesh sieve, resulting in G/GluP with an average particle size of less than 0.7 mm. 

### 2.5. Synthesis of O-CMCS/OHA Powder without and with the Crosslinker Pigment and Characterization of O-CMCS/OHA Hydrogels

For the preparation of O-CMCS/OHA powder without the crosslinker pigment, ratios of O-CMCS:OHA used were 1:1, 1:0.75, 1:0.5, and 1:0.25. For the O-CMCS/OHA powder with the crosslinker pigment, ratios of O-CMCS:OHA were 1:1, 1:0.75, and 1:0.5, with a fixed amount of 2.0 mg of the crosslinker pigment G/GluP. The different formulations of O-CMCS/OHA powder with varying concentrations of OHA were prepared to investigate the effect of OHA on the gelation rate of the powder. The powders were homogenized in a mortar with liquid nitrogen for approximately 3 min. Subsequently, 0.2 g of each powder without the crosslinker pigment (O-CMCS/OHA) and with the crosslinker pigment (O-CMCS/OHA-G/GluP) were transferred to Petri dishes and mixed with 3 mL of ultrapure water to obtain non-crosslinked hydrogel (O-CMCS/OHA HG) and crosslinked hydrogel (O-CMCS/OHA-G/GluP HG). The composition of the prepared formulations is detailed in Table 1.

### 2.6. Characterization

The organoleptic properties and chemical composition of the O-CMCS, OHA, G/GluP, and O-CMCS/OHA powder were evaluated. To characterize the chemical composition, Fourier Transform Infrared (FTIR) spectroscopy was employed. The spectra of the samples were recorded over a range of 650 to 4000 cm^−1^, with 16 scans at resolution of 4 cm^−1^. These measurements were conducted using a Spectrum 400 FT Mid-IR spectrometer by PerkinElmer (Waltham, MA, USA).

The hydrogels, both with and without the crosslinker pigment (O-CMCS/OHA HG and O-CMCS/OHA-G/GluP HG), prepared by mixing 0.2 g of each powder in 3 mL of ultrapure water, were rheologically characterized using a HAAKE™ MARS™ rheometer (Thermo Fisher Scientific, Waltham, MA, USA), equipped with a PP35TiL rotor with plate–plate geometry and a gap of 0.5 mm. A frequency ramp was conducted from 0.1 to 100 Hz. Following this, measurements were taken under constant frequencies of 8.5 Hz and 10 Hz for a duration of 15 min at a temperature of 37 °C.

## 3. Results and Discussion

### 3.1. Organoleptic Properties and FTIR of Hydrogel Precursors

#### 3.1.1. O-Carboxymethyl Chitosan (O-CMCS)

**Figure 1:**
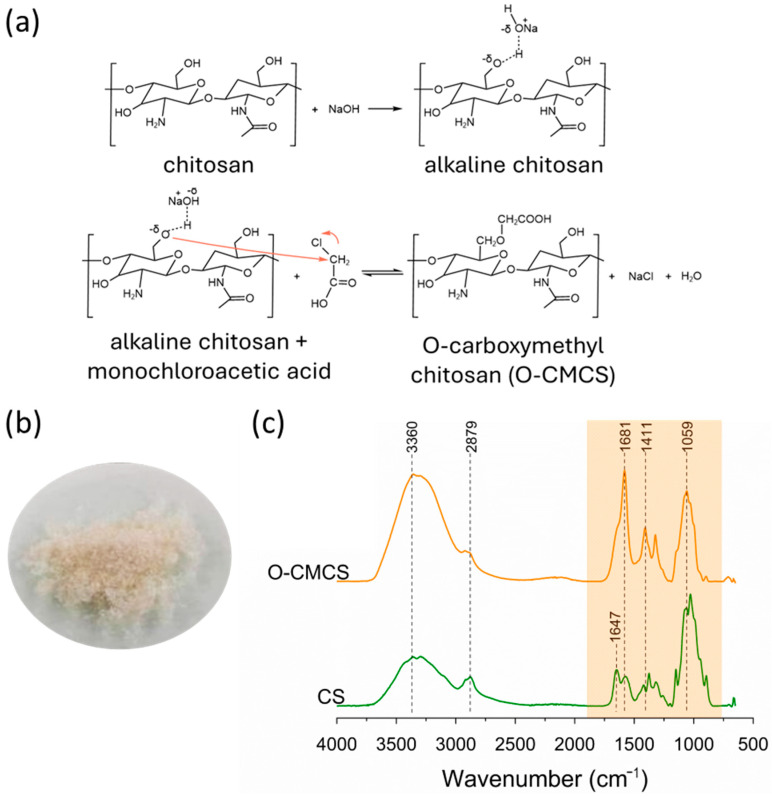
Reactions involved in the synthesis of O-CMCS (**a**), visual presentation of O-CMCS (**b**), and FTIR spectra of CS and O-CMCS (**c**).

The reactions involved in the synthesis of O-CMCS are illustrated in Figure 1a. The synthesized O-CMCS appeared as yellow-brownish (Figure 1b) and was characterized as hard, rough, and odorless. Figure 1c shows the FTIR spectra of chitosan (CS) and O-CMCS. In the FTIR spectrum of CS, the absorption peak near 3360 cm^−1^ is attributed to the stretching vibration of O-H and N-H bonds; the weak broadband near 2879 cm^−1^ corresponds to the C-H bond vibration, and the absorption peak of the amino group C2 of chitosan appeared at 1644 cm^−1^. In the FTIR spectra of O-CMCS, the absorption peaks around 1581, 1411, and 1059 cm^−1^ correspond to the asymmetric stretching vibrations of the carboxyl group, confirming the successful carboxymethylation of chitosan [35,59,60,61]. Thus, the FTIR results conclusively indicate the successful synthesis of O-CMCS.

#### 3.1.2. Oxidized Hyaluronic Acid (OHA)

**Figure 2:**
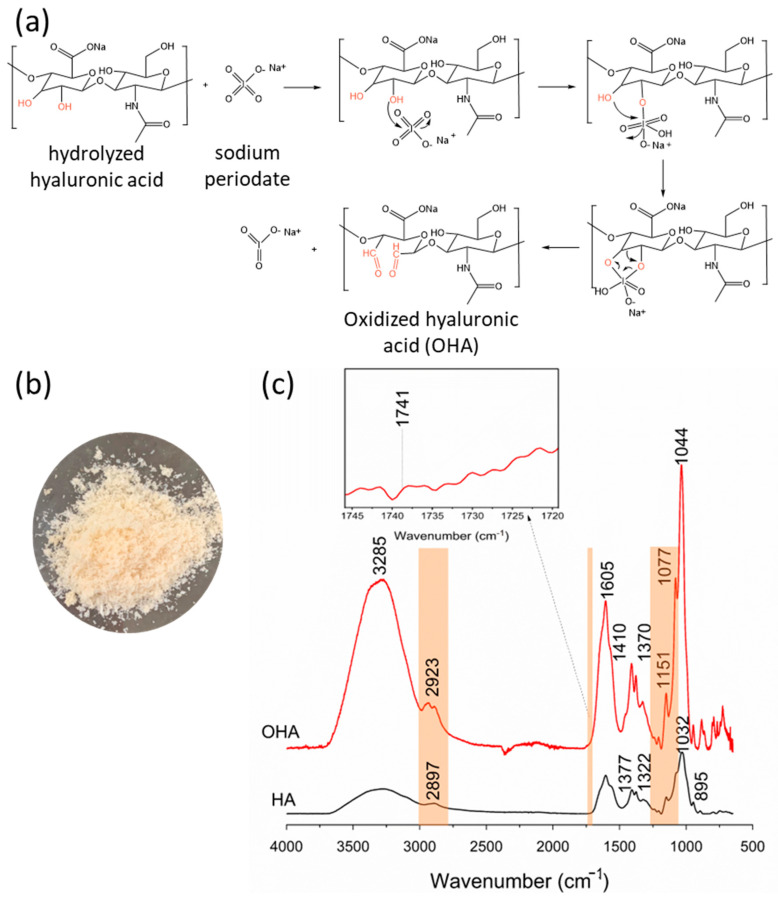
Reactions involved in the synthesis of OHA (**a**), visual presentation of OHA (**b**), and FTIR spectra of HA and OHA (**c**).

Figure 2a illustrates the reaction between hydrolyzed hyaluronic acid and sodium periodate, resulting in the formation of oxidized sodium hyaluronate (OHA). The synthesized OHA in powder form appeared yellowish (Figure 2b) and was identified as a hard, rough, and odorless material. The FTIR spectrum of HA (Figure 2c) displays absorption bands at 3285 cm^−1^, attributed to the stretching of the N-H group in combination with C=O. The band at 2897 cm^−1^ indicates the stretching of the C-H group, while a sharp peak at 1605 cm^−1^ corresponds to the asymmetric stretching of C=O. Additionally, stretching of C-N is observed at 1322 cm^−1^. The band at 1377 cm^−1^ is attributed to the deformation of the CH, CH_2_, and CH_3_ groups. The peak at 1032 cm^−1^ corresponds to the stretching of the C-O-C, C-O, and C-O-H groups. The peak at 895 cm^−1^ is attributed to the presence of the C-O-C group and the deformation of carbonyl and hydroxyl groups [62,63].

In the FTIR spectrum of OHA (Figure 2c), higher-intensity peaks and bands are observed, with no significant differences compared to HA. There is a peak at 2923 cm^−1^, indicating stretching of C-H bonds, and a peak at 1151 cm^−1^, corresponding to glycosidic bonds (C-O-C, O bridge), consistent with findings reported by Li et al. [64]. However, at 1741 cm^−1^, a shoulder appears corresponding to the symmetric vibration of the C=O group in the aldehyde portion, validating the modification reaction of hydrolyzed hyaluronic acid (HA) and the production of OHA.

According to Nguyen et al. [65], the challenge in detecting the aldehyde group signal in the OHA chain is due to the formation of hemiacetals. However, in the synthesized OHA sample, the signal of the aldehyde group C=O was visible, confirming the oxidation reaction of HA by sodium periodate. Similar results have been obtained by other researchers, underscoring the effectiveness of the method used [62,66].

#### 3.1.3. Crosslinker Pigment (G/GluP)

**Figure 3:**
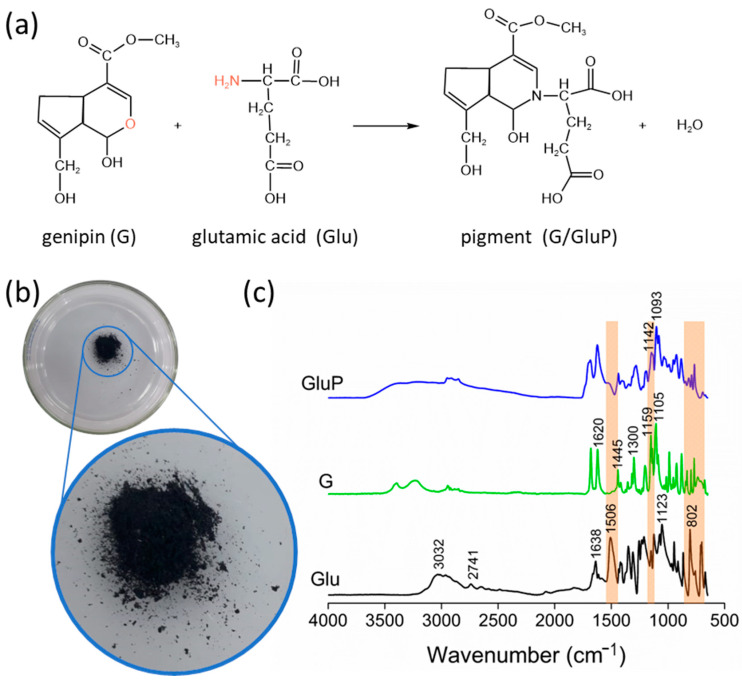
Visual presentation of crosslinker pigment G/GluP (**a**), reactions involved in the synthesis of G/GluP (**b**), and FTIR spectra of Glu, G, and G/GluP (**c**).

The reaction of genipin (G) with glutamic acid (Glu) to obtain the crosslinking pigment (G/GluP) is shown in Figure 3a. The crosslinking pigment G/GluP exhibits a characteristic blue color (Figure 3b). According to the FTIR spectra (Figure 3c), the absorption peak at 3032 cm^−1^ corresponds to the vibrations of the primary amine group (–NH_2_) present in glutamic acid (Glu) [67]. Peaks at 1506 cm^−1^ and 750–850 cm^−1^ are attributed to the angular deformation of N-H in the primary amine of Glu [67,68,69]. The peak at 2741 cm^−1^ represents the stretching vibration of C-H in Glu. The strong absorption peak at 1638 cm^−1^ indicates the stretching vibration of C=O in the carboxylic acid group of Glu. The presence of C-O stretching is confirmed by the peak at 1123 cm^−1^, corresponding to the carboxylic acid. The peak at 802 cm^−1^ is attributed to the planar deformation of O-H [70,71,72,73].

The spectrum of genipin (G) shows a peak at 1620 cm^−1^, related to out-of-plane stretching vibrations of C=C. The peak at 1105 cm^−1^ can be attributed to C-O stretching vibrations of the primary alcohol in the genipin structure [74,75]. Additionally, asymmetric stretching of C-O-C is observed at 1300 cm^−1^ and methyl ester bending at 1445 cm^−1^. The absorption at 1159 cm^−1^ corresponds to cyclic ether vibrations [76].

In the spectrum of the G/GluP, partial disappearance of the bands at 1506 cm^−1^ and 750–850 cm^−1^, corresponding to the N-H bending of the primary amine of glutamic acid (Glu), was observed. This indicates the formation of the pigment through the reaction between the primary amine of glutamic acid and the ether group of genipin, resulting in the formation of a tertiary amine and the elimination of the primary amine of Glu. Additionally, the peak at 1142 cm^−1^ can be attributed to the overlap of peaks at 1159 cm^−1^ and 1123 cm^−1^, related to cyclic ether vibrations of genipin and the C-O stretching of the carboxylic acid group in Glu. It is important to note a reduction in this peak, indicating a decrease in cyclic ether, as part of the pigment formation process.

#### 3.1.4. O-CMCS/OHA and O-CMCS/OHA-G/GluP Powders

**Figure 4:**
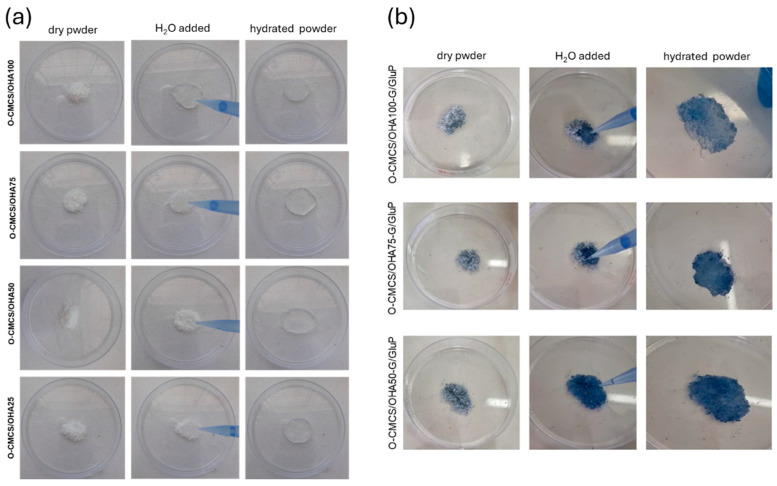
Visual presentation of powders without crosslinker (O-CMCS/OHA100, O-CMCS/OHA75, O-CMCS/OHA50, and O-CMCS/OHA25) (**a**) and with crosslinker (O-CMCS/OHA100-G/GluP, O-CMCS/OHA75-G/GluP, and O-CMCS/OHA50-G/GluP) (**b**).

A visual presentation of the powders and hydrogel formation, with different ratios of O-CMCS and OHA with and without G/GluP, is shown in Figure 4. The powder without the crosslinker pigment (O-CMCS/OHA) appeared white. The powder with the crosslinker pigment (O-CMCS/OHA-G/GluP) exhibited a characteristic blue coloration due to the pigment.

The powders O-CMCS/OHA and O-CMCS/OHA-G/GluP were transformed into hydrogels by adding 3 mL of ultrapure water to 0.2 g of each powder. Immediate hydrogel formation was observed upon the addition of water to the O-CMCS/OHA-G/GluP powder in a 1:1 ratio (O-CMCS/OHA100-G/GluP), indicating that a higher amount of OHA and the presence of the crosslinking pigment resulted in a shorter gelation time. Consequently, the hydrogel prepared with the O-CMCS ratio 1:1 was selected for rheological characterization.

**Figure 5:**
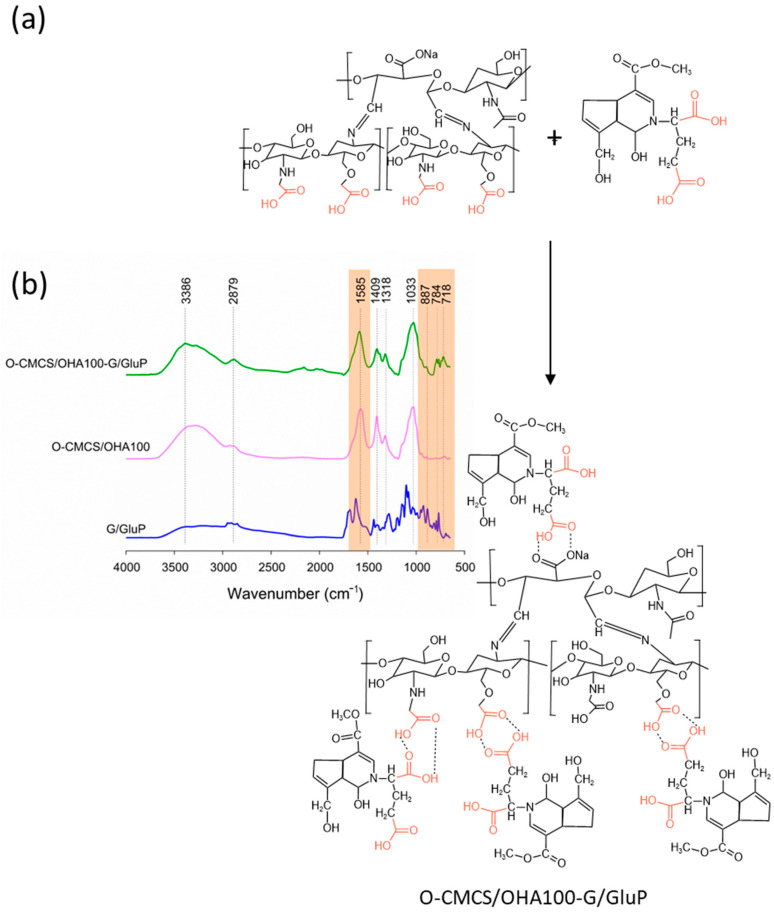
Reactions involved in the synthesis of crosslinked powder (**a**) and FTIR spectra of G/GluP and powders without crosslinker (O-CMCS/OHA100) and with crosslinker (O-CMCS/OHA100-G/GluP) (**b**).

The freeze-dried hydrogels with the formulation containing O-CMCS/OHA powder in a 1:1 ratio of O-CMCS to OHA, both without and with the crosslinker pigment (O-CMCS/OHA100 HG and O-CMCS/OHA100-G/GluP HG), were subjected to FTIR spectroscopy (Figure 5) to confirm the formation of the Schiff base. According to the literature, the formation of the Schiff base, due to the reaction between the amino groups of O-CMCS and the aldehyde groups of OHA, results in the gelation process of the powder [62].

Analyzing the spectra in Figure 5, all O-CMQ/OHA hydrogels exhibit a peak around 1585 cm^−1^. Agreeing with Nguyen, Nguyen, Tran, Nguyen, Huynh, and Vo Van [65], this peak near 1600 cm^−1^ can be attributed to C=N stretching, indicating the presence of the Schiff base bond (–CH=N–). The spectrum of the crosslinker pigment G/GluP exhibits amine bands in the region of 750–850 cm^−1^, present in the O-CMCS/OHA100-G/GluP HG, aligning with findings from [77]. The crosslinking reaction of the O-CMCS/OHA powder with the crosslinker pigment (G/GluP) is shown in Figure 5b.

### 3.2. Rheological Properties of Hydrogels

**Figure 6:**
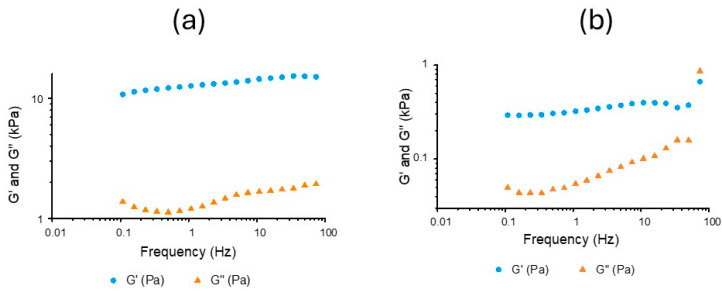
Curves of *G*′ and *G*″ as a function of frequency for the hydrogel prepared with the crosslinker pigment (O-CMCS/OHA100-G/GluP) (**a**) and without the crosslinker pigment (O-CMCS/OHA100) (**b**).

The hydrogel prepared with the powder O-CMCS/OHA100-G/GluP appears solid throughout the analyzed frequency spectrum, with *G*′ being higher than *G*″, as can be seen in Figure 6a. This is due to the presence of the G/GluP crosslinker pigment, which can be noticed when analyzing the *G*′ and *G*″ curve as a function of frequency for the system without the presence of this crosslinker (see Figure 6b). It can be seen that it takes some time (inverse frequency) for the system to reach the gel point.

**Figure 7:**
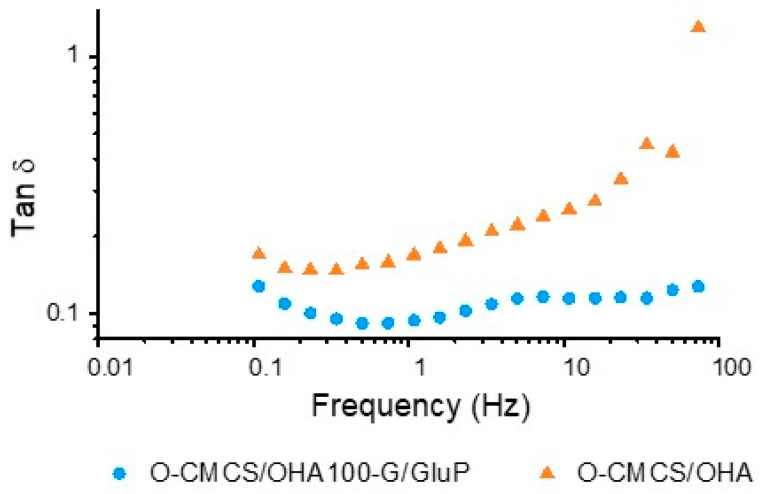
Curve of tan *δ* as a function of frequency for the O-CMCS/OHA100 hydrogel crosslinked with the G/GluP pigment (O-CMCS/OHA100-G/GluP HG).

Another interesting aspect of the presence of the G/GluP crosslinker pigment in the system is that not only a considerable increase in *G*′ values across the entire frequency spectrum exists but also a greater distance between *G*′ and *G*″, which allows lower values for the loss tangent (see Figure 7), generating more stability to the gel and ensuring it remains in the gel state for a longer time, which is characterized by tan⁡δ < 1.0. This indicates that the hydrogel′s properties will likely be maintained in the dynamic environment of the body. These results are consistent with those presented by Stapleton, Steele, Wang, Lopez Hernandez, Yu, Paulsen, Smith, Roth, Thakore, and Lucian [10], who developed a supramolecular hydrogel formed by combining dodecyl (C12)-modified hydroxypropylmethylcellulose (HPMC), known as HPMC-C12, and polymeric nanoparticles composed of poly (ethylene glycol)-b-poly (lactic acid) (PEG-PLA).

**Figure 8:**
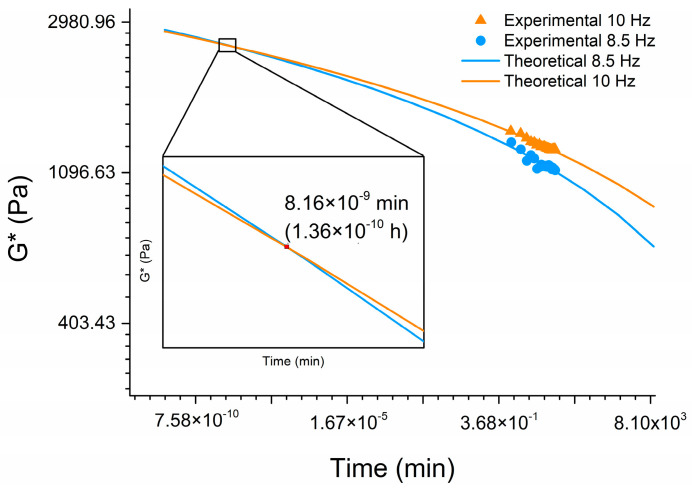
Curves of the complex modulus as a function of frequency for the O-CMCS/OHA100 hydrogel crosslinked with the G/GluP pigment (O-CMCS/OHA100-G/GluP HG).

Since the system with the G/GluP crosslinker pigment presented solid behavior throughout the analyzed frequency spectrum, it is not possible to use the criterion for determining the gel point or time by crossing the *G*′(w) and *G*″(w) curves nor the Winter–Chambon criterion [78] because tan⁡δ depends upon frequency since $t>t_{g}=0$, as can be seen in Figure 8. So, two frequencies in the range in which *G*′ and *G*″ are independent of frequency were used to observe the variations in *G*′(t) and *G*″ (t) and, therefore, G*(t) for 15 min. The gel time, $t_{g}$, will be the time where the curves relating to the two constant frequencies intersect.

The behavior of G*(t), the complex modulus, of the system with the G/GluP crosslinker pigment at frequencies of 8.5 Hz and 10 Hz for 15 min is shown in Figure 8. The aim for plot G*(t) using two constant frequencies was to use a more robust strategy for gel point identification instead of the strategy based on the intersection of elastic and viscous moduli, which has been repeatedly used in the literature but provides only a rough approximation. To overcome this limitation, a more rigorous approach based on multifrequency experiments (Winter–Chambon criterion) combined with Moura et al.’s [79] approach, using G*(t) instead of tan (t), was used. In this approach, the intersection point of at least two G*(t) curves at two constant frequencies determines the gel point.

The logarithmic adjustment of the data allowed determining the gel time (*t_g_*) of the system with the G/GluP crosslinker pigment as being 1.36 × 10^−10^ h (Figure 8), in other words, *t_g_* can be taken as zero. These data confirm the initial statement that the system with the G/GluP crosslinker pigment has always behaved like a solid, meaning *t* > *t_g_*. For use as a bleeding stopper, the immediate gelation of the system with the G/GluP crosslinker pigment in the presence of a liquid medium (water), a *t_g_* approximately equal to zero is extremely important.

For other uses, especially via injection, in addition to preserving the mechanical resistance observed by maintaining the initial viscoelastic character over a wide range of frequency or time, it is important to observe the flow behavior of the gel containing the G/GluP crosslinker pigment. For this purpose, the behavior of complex viscosity was observed in the frequency range used.

**Figure 9:**
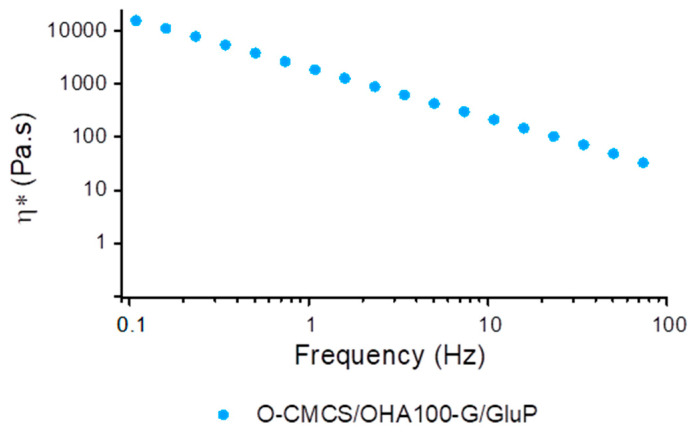
Curve of the complex viscosity as a function of frequency for the O-CMCS/OHA100 hydrogel crosslinked with the G/GluP pigment (O-CMCS/OHA100-G/GluP HG).

In Figure 9, we can observe a pseudoplastic behavior with a high degree of pseudoplasticity, which guarantees a good perspective for the handling and spreading of the gel as well. Additionally, given the ability to drastically reduce viscosity at initial frequencies and maintain an approximately constant value in the final frequency range and due to the close relationship between $\eta^{*}(\omega )$ and $\eta \left(\dot{\gamma }\right)$, the Cox–Merz rule guarantees whether there is good injectability of the hydrogel prepared with the O-CMCS/OHA100-G/GluP powder.

Based on the results of the rheological analysis, the O-CMCS/OHA100-G/GluP hydrogel exhibited pseudoplastic behavior, which facilitates the manipulation, spreading, and injectability of the hydrogel, making it suitable for practical applications in clinical settings.

## 4. Conclusions

In this study, O-CMCS and OHA were successfully synthesized and demonstrated their effective integration into hydrogel formulations using the G/GluP crosslinker pigment. The FTIR analysis confirmed effective chemical modifications and the formation of the target compounds. The resulting hydrogels presented immediate gelation upon contact with water, an important feature for their application as physical barriers to prevent post-surgical adhesions. Rheological evaluations revealed that the crosslinked hydrogels (O-CMCS/OHA-G/GluP) displayed significantly higher elastic moduli (*G*′), indicating superior gel formation and mechanical stability compared to non-crosslinked hydrogels. The inclusion of the G/GluP crosslinker enhanced gel stability by increasing the separation between *G*′ and *G*″ and achieving a lower loss tangent (tan *δ* < 1.0), suggesting robustness under dynamic physiological conditions. The rapid hydration and gelation properties further emphasize their potential as effective physical barriers. Additionally, the O-CMCS/OHA-G/GluP hydrogel demonstrated rapid self-healing, enabling efficient application via spraying or spreading, along with tissue adherence for local retention over clinically relevant time frames and viscoelasticity to allow tissues and organs to move freely, thereby preventing adhesions. Moreover, the hydrogel proved to be cost effective and scalable, making it a promising solution for clinical applications aimed at preventing post-surgical adhesions.

## Figures and Tables

**Table 1:** Composition of the powder formulations prepared in this study.

Sample	O-CMCS (mg)	OHA (mg)	G/GluP (mg)
O-CMCS/OHA100	100.0	100.0	-
O-CMCS/OHA75	100.0	75.0	-
O-CMCS/OHA50	100.0	50.0	-
O-CMCS/OHA25	100.0	25.0	-
O-CMCS/OHA100-G/GluP	100.0	100.0	2.0
O-CMCS/OHA75-G/GluP	100.0	75.0	2.0
O-CMCS/OHA50-G/GluP	100.0	50.0	2.0

## Data Availability

The original contributions presented in the study are included in the article, further inquiries can be directed to the corresponding authors.
